# Numeric Databases in Chemical Thermodynamics at the National Institute of Standards and Technology

**DOI:** 10.6028/jres.094.004

**Published:** 1989

**Authors:** Malcolm W. Chase

**Affiliations:** Chemical Thermodynamics Division, National Institute of Standards and Technology Gaithersburg, MD 20899

**Keywords:** chemical thermodynamics, chemical thermodynamics data, data files, enthalpy, evaluated data, heat capacity, JANAF Thermochemical Tables, numeric databases, phase transitions, vapor pressure

## Abstract

During the past year the activities of the Chemical Thermodynamics Data Center and the JANAF Thermochemical Tables project have been combined to obtain an extensive collection of thermodynamic information for many chemical species, including the elements. Currently available are extensive bibliographic collections and data files of heat capacity, enthalpy, vapor pressure, phase transitions, etc. Future plans related to materials science are to improve the metallic oxide temperature dependent tabulations, upgrade the recommended values periodically, and maintain the bibliographic citations and the thermochemical data current. The recommended thermochemical information is maintained on-line, and tied to the calculational routines within the data center. Recent thermodynamic evaluations on the elements and oxides will be discussed, as well as studies in related activities at NIST.

## Introduction

The Chemical Thermodynamics Data Center provides the chemical process industry with critically evaluated thermodynamically consistent data which can be used to establish the equilibrium constants and enthalpies of reaction for important chemical reactions. These critically evaluated data are used in the design and interpretation of research in physics, chemistry, biochemistry, geochemistry, environmental sciences, metallurgy, and other fields where chemical interpretations are important. The center provides this data describing the change in the chemical properties of substances as well as bibliographic reference services in thermochemistry.

In characterizing the thermodynamic properties of chemical species, the primary effort involves the study of inorganic species and their aqueous solutions. Additional efforts are also directed at the examination of organic species and biological systems. In the area of inorganic chemistry, this data center has the responsibility for two major publications: The NBS Tables of Chemical Thermodynamic Properties [1] and the JANAF Thermochemical Tables [2]. The former publication deals with the property values at 298.15 K for 92 elements and their compounds while the latter presents temperature-dependent values for 49 elements and some of their compounds.

Currently, the data center staff is involved in extending and upgrading the information contained in these two publications. Although the projects which led to these publications as a product are continuing, they are following a different approach from that used in the past. Part of this change results from the merger of the two above-mentioned projects. In addition there are many cooperative ventures in which the data center is involved. These also require a slightly different approach to the evaluation process. The following discussion will mention the accessibility of the NIST recommended data and the procedures being followed for the study of the elements and the oxides.

## Accessibility of Thermodynamic Data

The information contained in the previously mentioned two publications is easily available to users in hard copy. Both publications appeared as supplements to the Journal of Physical and Chemical Reference Data and are readily available from the American Chemical Society. Additionally, magnetic tape versions of both publications are available from the Office of Standard Reference Data at the National Institute of Standards and Technology. Both data sources are also available through an on-line commercial vendor, CAS-STN. The databases are called NBSTHERMO and JANAF, respectively. Thus, in principle, this thermodynamic information is quickly and easily obtainable for any users.

Within the NIST Chemical Thermodynamics Data Center, these resources are accessible via an on-line system. This has been developed to tie together the information from these databases with many of the programs used in the critical evaluation of data. Current data evaluation efforts are in progress for the JANAF Thermochemical Tables, the NBS Tables of Chemical Thermodynamic Properties, the CODATA Task Group on Chemical Thermodynamic Tables, as well as other cooperative efforts. It is necessary that each member of our staff have access to the centralized collection of this recommended information.

The NBS Tables are stored and retrieved on our HP-1000 system^1^ using commercially available database management software, IMAGE/1000 and ASK/1000. The database is searchable via the chemical formula, as it appears in the published hard copy [1]. The retrieval process provides up to six thermodynamic property values, namely, enthalpy of formation at *T*/K=0 and 298.15, and heat capacity, entropy, enthalpy, and Gibbs energy of formation, all at *T*/K=298.15.

The JANAF Thermochemical Tables are stored and retrieved using a locally developed search and retrieval system called SETKY-GETKY [3]. Searching the database is done via chemical formula (in the normal order) or chemical formula (in the Chemical Abstracts order) coupled with the physical state. The search returns the temperature-dependent thermal functions, as published in the hard copy [2]. Additional information includes the enthalpy of formation at 298.15 or 0 K, the value of the gas constant used, reference temperatures and pressures, transition temperatures, and origin and date of the table.

The main points to remember are that (1) the thermodynamic information for each species is obtainable during an interactive session at the computer terminal, (2) the thermodynamic information is centrally located on our minicomputer, and (3) the data evaluation programs used in our data center are tied into these databases programmatically.

## Element Study

Our efforts in the characterization of the thermodynamic properties of the elements illustrate our current evaluation approach. For our purposes the study of the elements involves the condensed phases (including all crystal, liquid, and amorphous states) and the gaseous phases (including the monatomic gas), any additional gas phase *n*-mers, and any positive and negative ions that exist.

The characterization of the gas phase molecules is very important in that they are necessary for the description of the vapor phase above any condensed phase elemental system. As an example, near the melting point of boron, the ratio of the vapor pressure of B_2_(g) to B(g) is approximately 0.000009. Thus, at this temperature or lower, the contribution of the dimer to the total vapor pressure is unimportant. However, in the case of sulfur at the normal boiling point, approximately 717 K, at least eight species exist in the vapor, with S_6_(g), S_7_(g), and S_8_(g) being the dominant species, contributing 95% of the vapor pressure at this temperature.

## Oxide Study

The same approach is used in the study of the oxides. We are concerned with the characterization of the condensed and gaseous phases, including the examination of the possibility of the existence of *n*-mers in the gas phase. Currently under examination are the alkaline earch metal oxides and the transition metal oxides (specifically, iron, nickel, and cobalt).

## Process for Evaluating Data

In general, the goal of the study of the thermodynamic properties of the elements is to generate temperature-dependent values for the heat capacity, enthalpy, Gibbs energy function, entropy, enthalpy of formation, Gibbs energy of formation, and the log of the equilibrium constant of formation. These tables also include various first-order transtition processes, such as solid-solid, fusion, sublimation, vaporization, etc. Second-order transitions must also be included, such as Curie points and superconducting temperatures.

For both the elements and the oxides, the evaluations are carried out using a multistep approach, with each step yielding a documented publication. Our users should be able to follow our work easily and see it on a shorter timetable.

The first step in this approach is to generate an annotated bibliography. The literature is constantly being scanned for data pertaining to the thermodynamic properties of the elements and the oxides. Relevant information is entered into bibliographic files, which are revised whenever new references are encountered. Each literature citation contains the following information:

1. author names
2. article title
3. journal citation
4. abstracting service reference
5. annotation indicating property studied

When a literature citation is found, the main emphasis is to enter the author names and journal citation. The remaining information will be added at a later time, if it is not readily available. The prime intent is to produce a complete listing of literature citations which is available to any user immediately. The remaining information, although useful and necessary for the final evaluation process, can be added later. Since these files are actively maintained on our computer, current versions may be printed immediately upon request.

In the second step, the relevant data are collected into tables and graphs, as references become available, and are stored in disk files actively maintained on our computer system. A combination of the bibliographic and numerical information is gathered together into a series of data summaries for easy appraisal of the quantity and quality of data available. At this point, the data (as stored in the disk files) can be directed through a series of programs to produce appropriate graphs which indicate visually the qualitative agreement (or the lack thereof) among the various temperature-dependent data sets. In addition, tabular summaries of the available studies are also maintained. At this point, no statement is made as to the reliability or goodness of any study. The intent is to know the studies which exist and what data are derived from them. The data summaries can also be distributed to interested users.

These “collection” phases are followed by an “evaluation” phase in which the available data are intercompared and, where possible, assessed against theory. In this phase, the recommendations are prepared. The process is described below for condensed phases and gaseous species.

## Condensed Phases

The generation of the thermal functions for the condensed phases of the elements and the oxides (that is, the heat capacity, enthalpy, entropy, and the Gibbs energy function) is based on a numerical integration of adopted or recommended heat capacity values. In general, these recommended heat capacity values are derived from a detailed analysis of many sources.

In order to accomplish this analysis, the literature search is for these prime sources of data: all heat capacity and enthalpy studies as a function of temperature—effects which cause changes in the heat capacity and discontinuities in the enthalpy must be included in the study. These include effects such as structural transitions. Curie temperatures, superconductive temperatures, and, in general, any first-or second-order transition.

After collecting the available literature, the data are extracted and summarized. The heat capacity and enthalpy are amenable to graphical comparisons. Typically there are four variations of graphs which are useful to consider:

1. *C_p_* vs *T* (all *T*)
2. *C_p_*/*T* vs *T*^2^ (for *T*<30 K), mainly for elements
3. (*C_p_−αT*)/*T*^3^ vs *T* (for *T* <30 K), mainly for elements
4. *H*(*T*)*−H* (298.15 K)/(*T*−298.15) (for *T*>298.15 K)

For the other data, such as temperatures and enthalpies of fusion, tables of the various studies are maintained for comparison purposes.

Currently, the emphasis is directed towards the hterature surveys, the data collection process, and the summary and display of the temperature-dependent information. Of course, data summaries are maintained for other information, such as the transition temperatures and enthalpies.

## Gas Phase Species

For the monatomic gases spectroscopic information is necessary for the evaluation and generation of thermochemical tables. Most important is the knowledge of the atomic energy levels, especially the low lying levels, the ionization potential, and the electron affinity. The atomic energy level information is obtained from the NIST Data Center on Atomic Energy Levels and is stored on-line so that calculations can be done at any time using a variety of calculational procedures.

For the *n*-mers, the information which is normally retrieved is the spectroscopic information dealing with the electronic energy levels and vibrational-rotational energy level information. Again, this information is maintained in data summary tables for easy comparison. As implied in the calculation of the thermodynamic properties for monatomic gases, the extent and type of data available for the *n*-mers will really determine the calculational pathway used. Significant difference can occur.

Also important is the vapor pressure information relating to sublimation, vaporization, decomposition, and reaction processes. Here, a plot of log *p* vs 1/*T* would be made. In this case, plots are useful not only to show the vapor pressure results but also to confirm the specification of the units (unfortunately the units are not always given in the literature). As with other data studies, a tabular listing of the available studies is also maintained.

## Previous Critical Evaluations

Also important in the collection of data is the Usting of the various critical evaluations that are already in existence. For example, if your interest is in aluminum, there are five to be considered! In most cases, the most recent evaluation would be preferred, since more data would be available. But there are cases in which evaluations differ. It is not always clear as to the cause of these differences, but they normally imply problems in the interpretation of the existing data.

## Summary

The prime recommended thermodynamic information generated within the Chemical Thermodynamic Data Center at NIST is available in hard copy from the American Chemical Society, in magnetic copy from the Office of Standard Reference Data at NIST, and interactively via our data center computer and CAS-STN.

As for the characterization of the thermodynamic properties of the elements and their oxides, the information is being made available in a series of sequential publications which will present annotated bibliographies, detailed data summaries, and evaluated thermochemical tables. During this sequential process, the same information is available through a series of active computer files. For those who need to be current as to new developments in the elemental and oxide thermodynamic information, we should have available annotated bibliographies, data summaries in terms of graphs and tables, and listings of available critical evaluations. More importantly, we can provide you with a detailed thermochemical table for the chemical species of interest. Currently, we have the ability to provide this information for roughly 50 elements and their oxides.

## Footnotes

1 Certain commercial equipment including computer software is identified in this paper in order to adequately specify procedures, experiments, and techniques used. Such identification does not imply recommendation or endorsement by the National Institute of Standards and Technology, nor does it imply that the materials, equipment, or software identified are necessarily the best available for the purpose.
